# Physical and radiographic features of degenerative retrolisthesis in Japanese female volunteers: an observational cohort study

**DOI:** 10.1038/s41598-023-27702-4

**Published:** 2023-01-09

**Authors:** Mutsuya Shimizu, Tetsuya Kobayashi, Hisashi Chiba, Issei Senoo, Kozaburo Mizutani, Kengo Sasai

**Affiliations:** 1 Department of Orthopaedic Surgery, Kyouritsu Hospital, 27-1N -W16, Obihiro, , Hokkaido 080-0046 Japan; 2 Department of Orthopaedic Surgery, Asahikawa Medical University, Asahikawa, Japan; 3 Furano Geriatric Health Services Facility, Furano, Japan

**Keywords:** Physiology, Anatomy, Medical research

## Abstract

Hundred and twenty four females with spondylolisthesis were divided into three groups (A group: anterolisthesis; P group: retrolisthesis; and AP group: antero-retrolisthesis), We reviewed their whole-spine radiographs and measured their standard sagittal parameters, including thoracic kyphosis (TK), pelvic incidence (PI), lumbar lordosis (LL), pelvic tilt (PT), and sacral slope (SS). The muscle strengths of the trunk flexor, trunk extensor, iliopsoas, and quadriceps were measured. Health-related quality of life was assessed using the Short Form 36-item Health Survey–physical component summary (SF-36 PCS). PI, SS, and LL-TK of participants in the P group were significantly lower than those in the A and AP groups (PI: P group vs. A group, p < 0.001, P group vs. AP group, p = 0.01), (SS: P group vs. A group, p = 0.001, P group vs. AP group, p = 0.003), (LL-TK: P group vs. A group, p < 0.001, P group vs. AP group, p = 0.049). TK of participants in the P and AP groups was greater than that of those in the A group. (P group vs. A group, p = 0.04, AP group vs. A group, p = 0.0025). The SF-36 PCS score in the P group was lower than that in the A and AP groups. (P group vs. A group, p = 0.004, P group vs. AP group, p = 0.012). The muscle strengths of the trunk flexor and trunk extensor and quadriceps in the P group were lower than those in the A groups. (Trunk flexor: P group vs. A group, p = 0.012), (Trunk extensor: P group vs. A group, p = 0.018), (Quadriceps: P group vs. A group, p = 0.011). In conclusion, female participants with degenerative retrolisthesis had a smaller PI and SS and a larger TK, along with decreased physical function and QoL scores than those with anterolisthesis.

## Introduction

Recently, whole spinal alignment in the sagittal plane has been emphasized as an important treatment modality for managing spinal diseases as it improves the quality of **life (QoL) and alleviates the disease condition^1–4^. Progression of spinal malalignment affects the adjacent tissues and joints, which worsens with increasing age. Spondylolisthesis is one of the most common findings observed in the sagittal plane among the elderly population^5^. Based on the direction of the slip in the sagittal plane, degenerative spondylolisthesis is divided into anterolisthesis, retrolisthesis, and antero-retrolisthesis.

Among these three groups, Barry et al. and Roussouly et al. reported that retrolisthesis is one of the compensatory mechanisms for maintaining sagittal balance in patients with advanced spinal malalignment^6,7^.

Recently, Jeon et al. reported that among 269 patients with degenerative spondylolisthesis, 106 (39.4%) had pure retrolisthesis^8^. Furthermore, Mihara et al. reported in a cohort study that lumbar retrolisthesis was observed in 41% of the study population and thus stated that the incidence of retrolisthesis can be considered as high^9^. Therefore, retrolisthesis is not as rare as we used to believe it to be.

However, only a few studies have described the relationship between spinal parameters and retrolisthesis^8–10^.

Although radiographic evaluation is commonly used to assess degenerative spondylolisthesis, physical function and QoL should also be assessed as they are important factors to evaluate age-related spinal changes^11^.

However, the association of retrolisthesis with the muscle strength of the trunk and lower extremity and QoL has not been well-documented.

If retrolisthesis represents an advanced stage of spinal deformity, we hypothesized that QoL and trunk lower extremity muscle strength should be poor in the three groups.

Therefore, this study aimed to investigate the radiographic and physical characteristics of retrolisthesis among adult female participants with degenerative spondylolisthesis to determine whether the presence of retrolisthesis is associated with reduced QoL and muscle weakness.

## Methods

This study was a part of the Asahikawa observational study of Spinal Aging in a Prospective cohort (ASAP), which is an ongoing study being conducted in the town of Memuro in eastern Hokkaido, Japan.

The town of Memuro has a population of 18,000 inhabitants, and agriculture is their main occupation. In cooperation with the town of Memuro, we started this study in 1997.

In this study, we used the convenience non-probability method as the sampling method.

The initial recruitment of the cohort was done by sending invitations to rural residents of the area using the information in a resident ledger. The participants were females over 40 years of age who were included in the ASAP study.

Details of the spine screening program were explained to the participants, and imaging and physical examinations were performed after obtaining informed consent from all participants.

Because muscle strength measurements were performed 2011 onwards, only participants from 2011 to 2020 were included in the study.

Whole spine radiographs were obtained with participants in a standing position who were instructed to stand in an upright relaxed posture with knees maximally extended and arms at their sides in the frontal view and supported by an armrest at the chest height in the lateral view.

**Figure 1:**
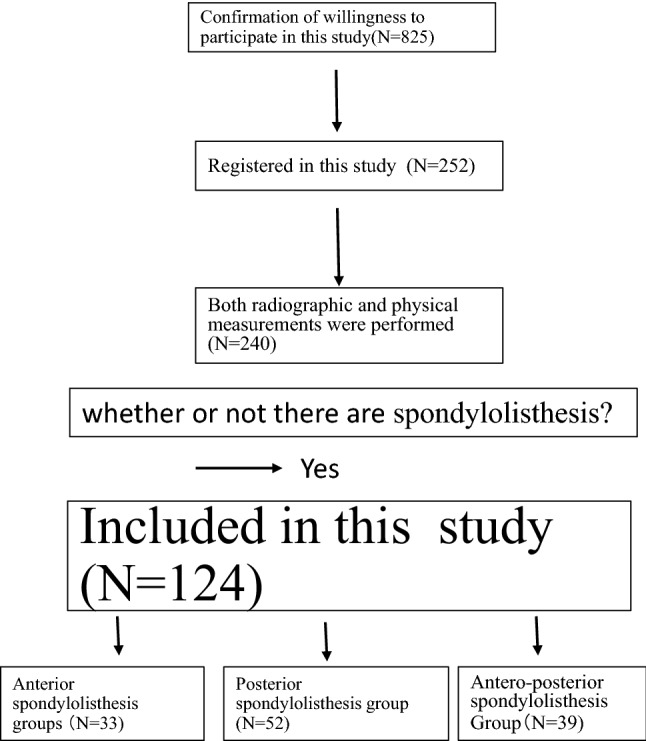
Flowchart of the enrolment process in our study. Finally, 33 participants in A group, 52 participants in P group, and 39 participants in AP group were eligible.

The inclusion criterion of this study was the presence of degenerative spondylolisthesis that was confirmed on radiograph (Fig. 1).

**Figure 2:**
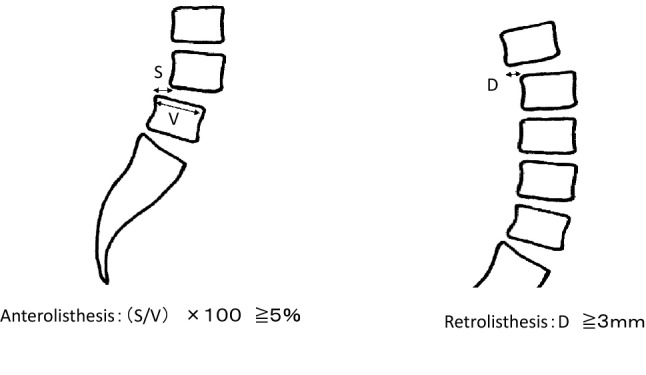
Definitions for anterior or posterior spondylolisthesis used in our study. The slip percentage was calculated by measuring the distance of sagittal translation between the adjacent vertebral endplates divided by the length of the inferior endplate, with a 5% slip diagnosed as anterior-degenerative spondylolisthesis, and the superior endplate, with a 3 mm slip diagnosed as retro- degenerative spondylolisthesis.

Anterolisthesis was defined as the translation of adjacent vertebral endplates divided by the length of the inferior endplate with a 5% slip, and retrolisthesis was defined as a backward slip of > 3 mm observed on a standing lateral lumbar radiograph^8,10^ (Fig. 2).

The exclusion criteria included patients with isthmic spondylolisthesis, those who underwent knee or hip replacement surgery and surgery of the spine, patients with neuromuscular diseases and tumor or infection of the spine, those who were unable to stand without assistance, and those with a Cobb angle of 30° or more in the coronal plane.

The participants were divided into three groups based on the direction of the slip: A group: anterior, anterolisthesis; P group: posterior, retrolisthesis; AP Group: anterior and posterior, antero-retrolisthesis).

Ethical approval for this study design was obtained from the Asahikawa Medical University Ethical Review Board (Approval No.: 372).

All methods were performed in accordance with the 1964 Helsinki declaration and its amendments or comparable ethical standards.

Written informed consent for both study participation and publication of identifying information/images in an online open-access publication was obtained from all patients.

### Radiographic measurements

**Figure 3:**
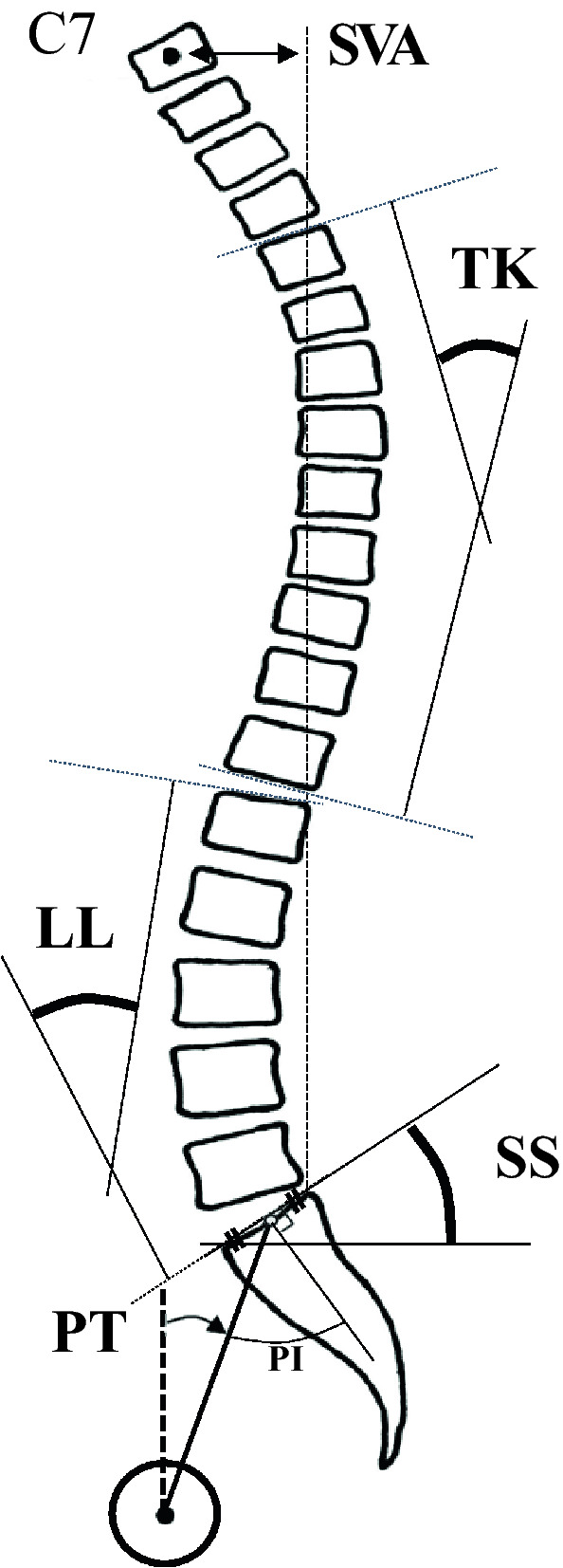
Radiographic measurements of the spinal sagittal parameters. Thoracic kyphosis (TK, the angle between the upper endplate of T4 and the lower endplate of T12). Lumbar lordosis (LL, the angle between the upper endplate of L1 and the lower endplate of S1). Sagittal vertical axis (SVA, distance between the C7 plumb line and the posterosuperior corner of S1). Pelvic tilt (PT, distance between the line through the center of the femoral head and midpoint of the sacral table and vertical reference).Sacral slope (SS, distance between the upper endplate of S1 and the horizontal line. Pelvic incidence (PI, a line perpendicular to the upper endplate of S1 and a line connecting the center of the femoral head to the center of the upper endplate of S1).

Radiographic measurements performed in the lateral view included TK (T4–T12, the angle between the upper endplate of T4 and the lower endplate of T12), LL (L1–S, the angle between the upper endplate of L1 and the lower upper endplate of the sacrum), SVA (C7–SVA, distance between the plumb lines through the posterosuperior corner of C7 and S1), PI (the angle between the line perpendicular to the middle of the sacral endplate and the line extending from the sacral midpoint to the center of the bicoxofemoral axis), SS (distance between the upper sacral endplate and the horizontal reference), and pelvic tilt (PT) (distance between the line through the center of the femoral head and midpoint of the sacral table and vertical reference) (Fig. 3).

### Physical measurements

All muscle strength measurements were performed as follows: both upper extremities were paired in front of the trunk and knee and hip joints were in 90-degree flexion in a chair-sitting position.

The pelvis and thighs were fixed with belts, and trunk flexion–extension movements were performed.

Measurement of the iliopsoas muscle was performed by placing a handheld dynamometer on the distal thigh and flexing it further from a 90-degree hip flexion position.

Measurement of the quadriceps muscle was performed by placing a handheld dynamometer on the distal lower leg and performing a further extension movement from a 90-degree flexion position of the knee joint. In order to avoid the effects of repetitive exercises, they were performed at intervals of at least 3 min.

**Figure 4:**
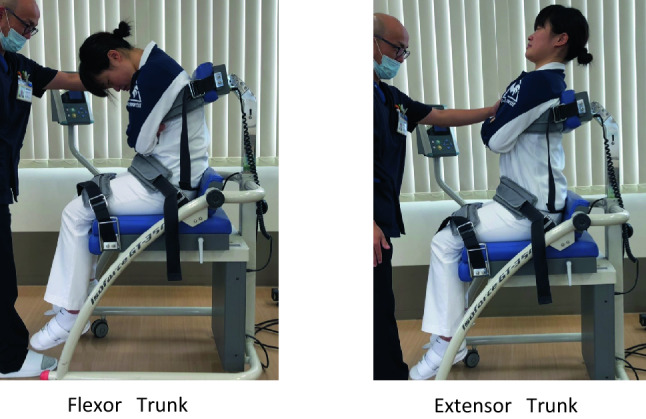
Physical measurements of the trunk muscle strength. The strengths of the trunk flexor and extensor muscles are measured using an isometric device (GT350, OG Giken Co., Japan).

Trunk flexor and extensor muscle strengths were measured using an isometric dynamometer (GT350, OG Giken Co., Japan) (Fig. 4).

**Figure 5:**
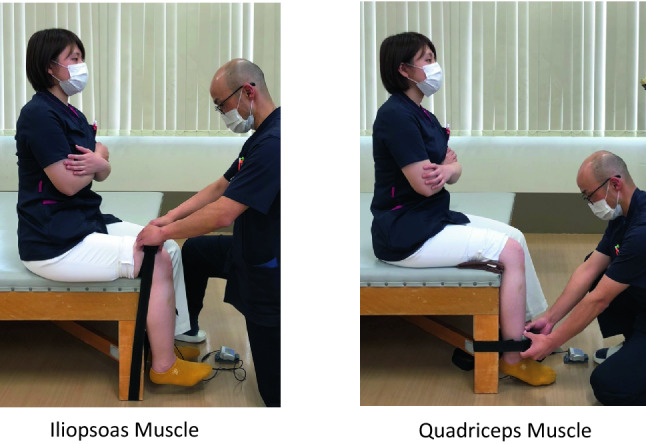
Physical measurements of the muscle strength of lower extremities. The muscle strengths of the quadriceps and iliopsoas were measured using an isometric device called a handheld dynamometer (μTasMF-01, Anima Co., Japan).

Quadriceps and iliopsoas muscle strengths were measured using an isometric device called a handheld dynamometer (μTasMF-01, Anima Co., Japan) (Fig. 5).

Each measurement was repeated at least three times for all participants, and the best scores were used.

### Clinical measurements

Clinical evaluations were also conducted by spine physicians and physical therapists, and the Short Form 36 health survey (SF36)–physical component summary (PCS) was used to assess the health-related QoL (HRQoL).

SF-36 is a widely used generic instrument that measures eight types of health constructs. SF-36 PCS is composed of four primary items, including the summaries of physical functioning (10 items), role limitation due to physical problems (4 items), pain (2 items), and general health (5 items). The scores range from 0 to 100, with a higher score indicating better HRQoL.

### Sample size calculation

In the previous assumptions, with a sample power of 80%, an effect size of 0.30, and a 2 –sided alpha level of 0.05 was used to calculate a sample size that provides statistically significant results.

The number of participants needed was 36.7 in each group and a total of 111 in the three groups.

### Statistical analysis

All statistical analyses were performed using SPSS Statistics, version 12 (IBM, Tokyo, Japan). All data are expressed as mean. The normal distribution of variables was verified using the Shapiro–Wilk test. Each continuous variable between each group was compared using the one-way analysis of variance with the Games–Howell post hoc test. A p-value of < 0.05 was considered statistically significant.

## Results

A total of 825 individuals were contacted about their willingness to participate, of which 252 registered for this study; however, only 240 of them were photographed. Finally, 124 female volunteers were included in the study.

The sample power was calculated based on the total number of participants in the three groups (124).The sample power obtained in this study was 0.846.

Table 1 shows the anthropomorphic characteristics and Tables 2, 3, 4 summarizes the level of the spondylolisthesis in the three groups (A group: anterior, anterolisthesis; P group: posterior, retrolisthesis; and AP Group: anterior and posterior, antero-retrolisthesis).

**Table 1:** Participants , anthropomorphic characteristics.

	A group	P group	AP group
No. of participants	33	52	39
AGE (years)	66.7	68.3	68.7
Height (m)	1.51	1.50	1.52
Weight (kg)	55.2	56.6	56.4
BMI (kg/m^2^)	24.2	25.2	24.4

A, anterior spondylolisthesis; P, posterior spondylolisthesis; AP, anteroposterior spondylolisthesis; BMI, body mass index.

**Table 2:** Level of spondylolisthesis in A group.

Level	Number
L3	8
L4	20
L3 + L4	4
L2 + L3	1

A, anterior spondylolisthesis.

**Table 3:** Level of spondylolisthesisin P group.

Level	Number
L1	11
L2	24
L3	6
L1 + L2	1
L2 + L3	3
L3 + L4	4
L1 + L2 + L3	1
L2 + L3 + L4	2

P, posterior spondylolisthesis.

**Table 4:** Level of spondylolisthesis in AP group.

Level	Number
L4(A) + L1(P)	16
L4(A) + L2(P)	7
L4(A) + L3(P)	1
L3(A) + L4(P)	1
L5(A) + L1(P)	2
L3 + L4(A) + L1(P)	3
L3 + L4(A) + L2(P)	1
L4(A) + L1 + L2(P)	2
L4(A) + L2 + L3(P)	3
L4(A) + L1 + L3(P)	2
L5(A) + L1 + L2(P)	1

A, anterior spondylolisthesis; P, posterior spondylolisthesis; AP, anteroposterior spondylolisthesis.

The radiographic and physical characteristics of participants in the three groups are presented in Tables 5 and 6.

**Table 5:** Differences of spinopelvic parameters of participants in the three groups.

Variable	A	P	AP	P Value		
				A vs P	A vs AP	P vs AP
Thoracic kyphosis (°)	24.3	34.3	34.4	0.04	0.025	0.934
Lumbar lordosis (°)	38.9	35.6	45.2	0.248	0.49	0.005
Lumbar lordosis- Thoracic kyphosis	15	1.3	10.8	< 0.001	0.165	0.049
Pelvic incidence (°)	57.7	47.9	53.7	< 0.001	0.3	0.01
Sacral slope (°)	30.9	23.6	30.8	0.001	0.796	0.003
Pelvic tilt (°)	27.4	24.2	23.2	0.545	0.505	0.99
Sagittal vertical axis (mm)	35.3	24.5	20.9	0.799	0.869	0.994

A, anterior spondylolisthesis; P, posterior spondylolisthesis; A + P, anteroposterior spondylolisthesis.

**Table 6:** Differences in physical function and QoL scores in the three groups.

Variable	A	P	AP	P value		
				A vs P	A vs AP	P vs AP
SF-36 PCS	50.6	46.5	49.8	0.004	0.9	0.012
**Variables**						
FL M (N/kg)	5.0	4.4	4.8	0.012	0.897	0.06
EX M (N/kg)	10	8.1	9	0.018	0.584	0.231
Quad M (N/kg)	0.53	0.45	0.52	0.011	0.94	0.051
Ilio M (N/kg)	0.33	0.28	0.32	0.092	0.952	0.198

A, anterior; P, posterior; AP, anterior and posterior; FL, Flexor Trunk; EX Extensor Trunk, anterior; Quad, quadriceps; Ilio, iliopsoas; M, muscle strength; QoL, quality of life; SF-36 PCS, Short Form 36 Health Survey–physical component summary.

Regarding the spinal parameters, the pelvic incidence (PI), sacral slope (SS), and lumbar lordosis (LL)-thoracic kyphosis (TK) of participants in the P group were significantly lower than those in the A and AP groups (PI: P group vs A group , p < 0.001, P group vs AP group, p = 0.01), (SS: P group vs A group, p = 0.001, P group vs AP group, p = 0.003,), (LL-TK: P group vs A group , p < 0.001, P group vs AP group , p = 0.049) .

TK of participants in the P and AP groups was greater than that of those in the A group (P group vs A group, p = 0.04, AP group vs A group, p = 0.0025).

Regarding HRQoL, the SF-36 PCS score of participants in the P group was lower than that of those in the A and AP groups (P group vs A group, p = 0.004, P group vs AP group, p = 0.012).

Regarding the physical function, the trunk flexor and trunk extensor and quadriceps muscle strength of participants in the P group was lower than that of those in the A group (Trunk flexor: P group vs A group , p = 0.012), (Trunk extensor : P group vs A group , p = 0.018), (Quadriceps: P group vs A group , p = 0.011).

## Discussion

This study demonstrated that the presence of retrolisthesis was associated with decreased HRQoL and trunk and lower extremities muscle strengths compared with anterolisthesis and antero-retrolisthesis.

In the present study, participants in the P group were characterized by a smaller PI and a larger TK; this finding is similar to that reported in a previous report by Jeon et al.^8^.

When the spinal deformity starts to decrease in LL^12^, which has an impact on the adjacent joints and tissues, a compensatory mechanism is activated.

However, smaller PI restricts the compensation of the pelvis, which may increase the impact on the thoracic spine, leading to an increase in TK and the progression of retrolisthesis.

Actually, in a cross-sectional study, Chung et al. reported that retrolisthesis increased with age and was observed in 75% of the elderly population aged over 65 years with a smaller PI.^13^.

Of note, other characteristic change in the parameter included a lower LL-TK is the P group compared with the other two groups. In a previous report by Yang et al., only LL-TK was reported as an index of HRQoL assessment, including TK^14^.

They stated that low LL-TK can be correlated with the disruption of the spine and pelvis, which can be associated with decreased QoL^14^. Furthermore, Zhou et al. indicated that LL-TK reflects the ongoing compensatory mechanisms and their relationship with the sagittal vertical axis (SVA). In their study, an LL-TK of > 10 indicated good surgical outcomes after an average of 3.2 years of follow-up^15^.

The SF-36-PCS score was significantly lower in the P group, indicating that ideal alignment is out of order and advanced spinal malalignment may act as a compensatory mechanism.

In addition, we showed that the muscle strength of the trunk and lower extremity was the lowest in participants in the P group.

Spinal malalignment was affected due to muscle activity, in particular, the spinopelvic and lower-extremity, because trunk and lower extremity muscles are attached to the spinal column and pelvis to stabilize the spine. Takemitsu et al. and Kobayashi et al. reported that sagittal malalignment of the spine is associated with decreased muscle strength^11,16^.

Furthermore, Enomoto et al. reported that on measuring muscle activity in lumbar kyphosis patients and canal stenosis patients using an electromyograph, it was noted that kyphosis patients had greater muscle activity in the resting position and the upper lumbar spine was at a level causing severe fatigue, which was consistent with the level where retrolisthesis was present in our study^17^.

Ferrero et al. evaluated the muscle characteristic in adult spinal deformity patients using 3D muscle reconstruction and found that fat infiltration was increased in all muscles when lumbar lordosis was reduced, and spinal erector group was the most affected among all muscle groups. Furthermore, they found that decreased LL affected muscle strength conditions as well as spinal radiographic alignment, including retrolisthesis^18^.

Another interesting observation in our study was the difference in spinal parameters, especially PI, between the participants in the P and AP groups.

Barry et al. reported that the progression of patients with degenerative spondylolisthesis starts with the anterior displacement of the vertebrae, which subsequently results in retrolisthesis of the spine; therefore, larger PI is a characteristic of patients with anterior or anteroposterior spondylolisthesis^19^. Similarly, Jeon et al. reported that PI is higher in patients with anteroposterior spondylolisthesis^8^.

However, it is unclear whether anterolisthesis precedes retrolisthesis or vice versa; therefore, further prospective longitudinal studies are warranted.

Our study has several limitations. First, the conditions of the intervertebral discs and facet joints, which are important diagnostic factors of spondylolisthesis, were not evaluated using either computed tomography or magnetic resonance imaging. Second, functional flexion/extension radiographs were not evaluated to diagnose spinal instability or joint hyperlaxity, which could be potential predictors of degenerative slip or instability. Third, the current study included only female participants who were relatively active and healthy. Sex differences in muscle aging have been reported; because we included only female participants, our results are not generalizable to both sexes.

In conclusion, our results imply that female participants with degenerative retrolisthesis had a smaller PI and LL and a larger TK, along with decrease in physical function and QoL scores than those with anterolisthesis and antero-retrolisthesis.

## Data Availability

The datasets generated and/or analyzed during the current study are available from the corresponding author on reasonable request.
